# Using Virtual Reality to Improve Outcomes Related to Quality of Life Among Older Adults With Serious Illnesses: Systematic Review of Randomized Controlled Trials

**DOI:** 10.2196/54452

**Published:** 2025-02-26

**Authors:** Bhagvat Maheta, Alexandra Kraft, Nickolas Interrante, Soraya Fereydooni, Jeremy Bailenson, Brian Beams, ​​Christina Keny, Thomas Osborne, Karleen Giannitrapani, Karl Lorenz

**Affiliations:** 1 Center for Innovation to Implementation VA Palo Alto Health Care System Palo Alto, CA United States; 2 College of Medicine California Northstate University Menlo Park, CA United States; 3 VA Center for Innovation to Implementation Chapman University Menlo Park, CA United States; 4 School of Medicine Yale University New Haven, CT United States; 5 Department of Communication Stanford University Stanford, CA United States; 6 Division of Geriatrics, Department of Medicine School of Nursing University of San Francisco San Francisco, CA United States; 7 Division of Geriatrics, Department of Medicine Veterans Affairs Medical Center University of California San Francisco, CA United States; 8 Department of Radiology School of Medicine Stanford University Palo Alto, CA United States; 9 Palo Alto Health Care System US Department of Veterans Affairs Palo Alto, CA United States; 10 Department of Primary Care and Population Health School of Medicine Stanford University Stanford United States

**Keywords:** virtual reality, serious illness, pain, anxiety, older adults, patient outcomes, systematic review, palliative care, hospice

## Abstract

**Background:**

Virtual reality (VR) has promise as an innovative nonpharmacologic treatment for improving a patient’s quality of life. VR can be used as an adjunct or treatment for many acute and chronic conditions, including serious illnesses.

**Objective:**

This systematic review aims to assess the current state of the literature of randomized controlled trials that use VR in patients with serious illnesses. Two secondary aims include assessing intervention components associated with improved quality of life and functional outcomes among older adults, as well as evaluating how well the randomized controlled trials adhere to consensus standards for VR research.

**Methods:**

We searched PubMed, Embase, and CINAHL for randomized controlled studies published at any time. We screened and accepted studies that reported outcomes related to patients’ quality of life, provided an immersive VR intervention, and included patients with serious illness. We narratively summarized key attributes of publications that shed light on study efficacy, generalizability, replicability, and clinical utility. All studies were assessed for study quality with the Cochrane Risk of Bias tool and for concordance with 8 recent consensus standards for VR research.

**Results:**

From the 12,621 articles searched in May 2024, a total of 24 (0.19%) studies met the inclusion criteria, and of these, 88% (21/24) reported an improvement in at least 1 patient quality of life outcome and 67% (16/24) had a high risk of bias. In 7 (n=24, 29%) studies, VR was used to provide distraction therapy to reduce pain. In total, 5 (n=24, 21%) studies included training, supervision, and assistance in VR use, which demonstrated improvements in patient quality of life–related outcomes. Of 24 studies, 9 (38%) included patients with stroke, 9 (38%) included patients with cancer, 4 (17%) included patients with cardiovascular disease, 1 (4%) included patients with chronic obstructive pulmonary disease, and 1 (4%) included patients who reported pain in hospital. In all 9 studies that included patients with stroke, the main purpose of VR was to improve mobility and strength; these studies had higher frequency and longer durations of VR use, ranging from 2 to 9 weeks, as compared to a VR use duration of <2 weeks for studies aiming to reduce pain or anxiety. Regarding consensus standards for VR research, 29% (7/24) of the studies adhered to all 8 criteria, and all studies (24/24, 100%) adhered to ≥5 criteria.

**Conclusions:**

Nascent evidence suggests VR’s potential in mitigating pain, anxiety, and depression and improving mobility among persons with serious illnesses. Most studies did not provide detailed information about unassisted or assisted use, suggesting that VR for older adults is currently most appropriate for observed settings with assistance available.

**Trial Registration:**

PROSPERO CRD42022346178; https://www.crd.york.ac.uk/prospero/display_record.php?RecordID=346178

## Introduction

### Background

More than 60% of Americans will face a period of significant disability before death, incurring a diminished quality of life from the impact of serious illness, including multimorbidity [1-3]. Serious illness is defined as a health condition with a high risk of mortality that either negatively impacts daily function or quality of life or strains a person’s caregivers [4]. Serious illnesses include those of indeterminate but limited prognosis and those that have a detrimental effect on the quality of life of patients and their caregivers. They encompass malignancy, organ failure, or frailty, a complex state of vulnerability that is often associated with dementia [4,5]. Serious illnesses are associated with burdensome symptoms and functional decline and include many leading causes of death, such as stroke, as well as multimorbidity [6]. Patients also face a significant financial burden because of medical treatment [7]. These conditions interfere with a person’s ability to engage in activities they once enjoyed and impose emotional and physical burdens on family members and other caregivers; these conditions are of growing importance with the population aging [4].

Functional outcomes are a useful adjunct to other quality of life–relevant measures to understand the impact of serious illness on a person’s life [8]. Activities of daily living (ADLs) include basic functions such as dressing, maintaining personal cleanliness, toileting, transferring, and eating. The impairment of 1 to 2 ADLs occurs in most Americans during the years before death, and ≥3 ADL impairments are associated with institutionalization. Important goals for patients include dying at home and maintaining cleanliness, both of which are abetted by functional status or require dedicated caregiving if adequate support is unavailable [9]. Evaluating functional outcomes in patients with serious illness is especially important because it directly reflects their quality of life and their ability to perform ADLs [10].

Virtual reality (VR) is one among emerging interventions (eg, smart technologies) with the potential to improve a patient’s quality of life, patient-reported outcomes, psychological outcomes, and functional outcomes for persons living with serious illness [11-14]. VR offers a computer-generated, stereoscopically rendered, 3D visual environment, often with complementary sound, which responds continuously to a patient’s movement. Immersive VR creates a psychological experience of treating the virtual simulation as a real experience. The sensory experience is thus distinct from nonimmersive reality, such as augmented reality that allows the participant to see computer-generated images superimposed on the real-world visual field [15,16]. Immersion and distraction therapies have been shown to be helpful in improving quality of life and functional outcomes. The use of VR in health care for serious illness care has been expanding to address pain, anxiety, and other needs in palliative care and hospice settings [17,18]. There is a need to understand the quality of research and VR’s efficacy, especially in randomized controlled trials (RCTs), as it applies to older, seriously ill adults.

### Objectives

Several recent reviews have explored content salient to our interest, although not in the specific context of applying VR to palliative care and serious illness [19-21]. We aim to determine the extent to which rigorous studies have focused on the rapidly growing population of older adults, the quality of that research, the extent to which it adheres to recently accepted VR research standards, and the characteristics of that research (eg, common outcomes) [22-24]. Two secondary aims include assessing the components of interventions in RCTs associated with improved quality of life and functional outcomes, as well as assessing the extent to which the current RCTs related to VR adhere to recommended consensus standards for high-quality VR research.

## Methods

### Protocol and Registration

Our review adhered to the PRISMA (Preferred Reporting Items for Systematic Reviews and Meta-Analyses) protocols statement and Enhancing the Quality and Transparency of Health Research guidelines [25,26]. We registered our protocol to the PROSPERO (CRD42022346178) [27].

### Eligibility Criteria

Included in this systematic review are studies published in peer-reviewed journals up through May 2024 that meet the following criteria organized by population, intervention, comparator, outcomes, timing, and setting framework (Textbox 1) [28]. Functional outcomes include patient-reported outcomes relating to the quality of life and patient-demonstrated outcomes (eg, arm curl, chair stand, back scratch, chair sit, reach, walk test, overall cognition, and memory) [29,30]. Biological intermediaries (eg, functional magnetic resonance imaging scans, cortisol levels, blood test values, and balance) that do not directly demonstrate patient function are not included as patient functional outcomes [31]. Eligibility criteria are described using the population, intervention, comparator, outcomes, timing, and setting framework (Textbox 1).

**Eligibility criteria using the population, intervention, comparator, outcomes, timing, and setting framework.**

**Population**

- Inclusion criteria: studies that include adult patients (aged ≥18 years) with serious illness, such as stroke, defined by a variable but limited prognosis and an important illness impact quality of life
- Exclusion criteria: studies that include pediatric or adolescent patients (aged ≤18 years) or patients without serious illness

**Intervention**

- Inclusion criteria: study participants must use some form of immersive virtual reality in the intervention arm.
- Exclusion criteria
  - Intervention description insufficient to describe immersiveness
  - Augmented or extended reality (ie, nonimmersive reality)
  - 2D screens (eg, Wii and Wii Fit [Nintendo], Xbox [Microsoft Gaming], and YouGrabber)

**Comparator**

- Usual care

**Outcomes**

- Inclusion criteria: patient quality of life–related outcomes, including patient-reported (eg, pain and anxiety) or patient-demonstrated (eg, arm curl, chair stand, back scratch, chair sit, reach, walk test, overall cognition, and memory) outcomes
- Exclusion criteria: only biological intermediaries reported as results (eg, functional magnetic resonance imaging scans, cortisol levels, blood test values, and balance)

**Timing: interventions with any follow-up period**

**Setting: any care setting (including in-patient clinics or outpatient and ambulatory care)**

### Selection of Sources for Evidence

Concepts included in the literature search were RCTs, VR, and serious illness as shown in Multimedia Appendix 1. We searched PubMed, Embase, and CINAHL for studies published at any time and identified 12,814 articles (n=10,799, 84.28% articles after duplicates were removed). During the title and abstract screening and full-text screening phases, 3 coauthor reviewers screened each study and were blinded to each other’s decisions (BM, AK, and SF). During the title and abstract screening, we resolved conflicts predominantly by a “gold standard” reviewer (KL or KG), occasionally by majority consensus. During the full-text screening, reasons for exclusion were identified. Only the gold standard reviewer (KL or KG) resolved conflicts during full-text screening. During full-text screening, if any systematic reviews met inclusion criteria, we added their included articles to the title and abstract screening phase. We used the Covidence (Veritas Health Innovation) software to generate a PRISMA diagram to track studies at each stage of the review (Multimedia Appendix 2 [25]) [32].

### Data Extraction

We built an abstraction form through an iterative process (Multimedia Appendix 3). All aspects of the included article interventions were recorded, including what the intervention entailed; the type of VR and media (eg, type of scenery or activity) used; any training, supervision, or assistance provided during VR use; the duration of sessions; frequency of sessions; and duration of intervention. The main outcomes collected were related to patient quality of life–related outcomes. Data abstraction for the included studies was done through Covidence with 2 reviewers per article. Two reviewers (BM, AK, or NI) abstracted each article independently and resolved all abstraction conflicts through a consensus discussion.

### Risk of Bias and Quality Assessment

Risk assessment for bias was done using the Cochrane Risk of Bias tool for RCTs [33]. Two reviewers (SF, AK, or BM) independently assessed the risk of bias in the domains of randomization, allocation concealment, blinding, accounting of patients and outcome events, and selective outcome reporting bias. Any disagreements were discussed by each of the 2 reviewers, and a consensus was reached.

### Synthesis of Results

We performed a narrative synthesis of the data abstracted due to the high degree of heterogeneity of the results, as each included article reported different forms of patient functional outcomes. We synthesized each VR-based intervention and extracted the purpose of VR within the intervention and whether there was any training, supervision, or assistance provided in the use of VR. To better understand the individual differences that may affect the efficacy of VR-based interventions, we looked at the average patient age, SD and range, type of serious illness, patient gender, patient ethnicity, and study location for each of the studies. We characterized each study’s adherence to recent consensus standards for high-quality VR research using the definitions below [22].

- Patient population: clear description of the study population (including inclusion and exclusion criteria)
- Clinical setting: appropriate setting for the VR treatment
- Control and randomization: justification for control and appropriate randomization
- Blinding and concealment of allocation: description of the method of allocation concealment
- End points: determination and justification of end points before the initiation of the study
- Study duration: determination and justification of the study duration before the initiation of the study; the study should be an a priori decision or hypothesis
- Presentation and analysis of results: clear presentation of results with appropriate statistical analysis
- Reporting the trial: reporting of the trial with a national or international registry (World Health Organization International Clinical Trials or ClinicalTrials.gov)

## Results

### Literature Selection

**Figure 1:**
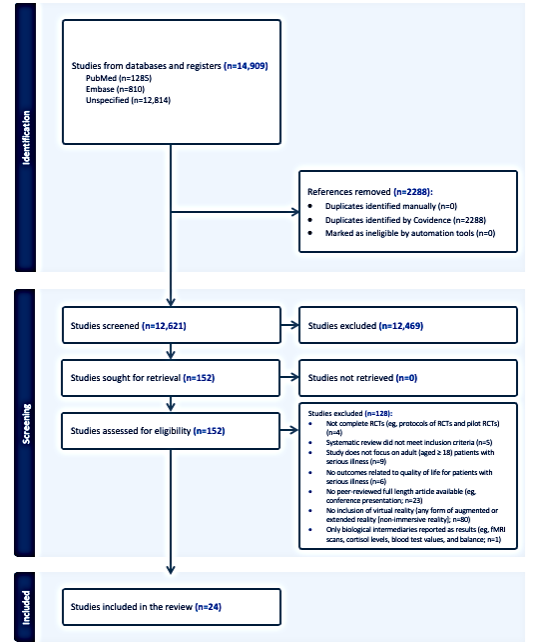
PRISMA (Preferred Reporting Items for Systematic Reviews and Meta-Analyses) flowchart. fMRI: functional magnetic resonance imaging; RCT: randomized controlled trial.

A total of 12,621 unique titles and abstracts were dual screened by coauthors as per the inclusion and exclusion criteria. A total of 152 full-text articles were retrieved and further assessed for eligibility. Of the 152 full-text articles reviewed, 128 (84.2%) studies were excluded for reasons noted in the PRISMA flow diagram (Figure 1 [34]). Finally, we included 24 total studies in our review [35-58], of which 21 (88%) reported an improvement in at least 1 patient functional outcome [35-43,45-48,51-58]. The included articles are summarized in Table 1.

**Table 1:** Summary of the included studies (n=24).

Study	Description of the intervention and media content	Control arm	Purpose of VR^a^ in the intervention	Training, supervision, or assistance provided in the use of VR	Patient-reported outcomes or patient-demonstrated outcomes
Bani Mohammad and Ahmad [35], 2018	The patients wore a head-mounted display with headphones and chose either deep sea diving or sitting on the beach. The VR exposure session ended at the peak time of morphine efficacy.	The patients were given morphine (same dose and timing as given to those in the intervention arm) with no additional intervention.	Distraction therapy to improve pain and anxiety in patients	The primary investigator remained near the participants during the VR session.	Statistically significant improvement in pain scores (VASb; P<.001) and anxiety levels (SAIc; P<.001)
Burrai et al [36], 2023	Participants used the Oculus Quest 2 HMD^d^ to observe specific virtual environments in a contemplative mode. The scenarios included 310 videos classified into 9 categories (eg, Africa, hills, rivers, lakes, and waterfalls). The audio content of scenarios had a background of nature sounds and soothing musical stimuli in high definition with panning stereo, and was listened to via earphones to ensure an immersive audiovisual experience with high-intensity multisensory immersion.	One group of patients received narrative medicine, where patients shared their experience with cancer. Another group of patients received standard care and served as the control arm.	Distraction from the stress of antiblastic therapy to reduce anxiety, fatigue, and pain	A nurse helped each participant position the VR headset and the joysticks. After immersion, the nurse removed the VR headset visor from the participant.	Statistically significant decrease in anxiety (P=.001) and fatigue levels (P=.001) but no statistically significant change in average pain levels (P=.31)
Chatterjee et al [37], 2022	The main requirement was to allow the patients to practice ADL^e^ completely or in part when placed within a suitable environment. The materials needed such as food items, kitchen gadgets, and coins were within reach of the patient when they were immersed in the environment. All the tasks were designed to be completed from a sitting position using a single handheld controller. The intervention was delivered 5 days a week for up to 2 weeks before the patient’s hospital discharge. The dose of the virtue treatment varied depending on the benefit and tolerability.	The patients received sham VR, where they were offered the same first VR session as the intervention arm and were subsequently offered the same VR session through the rest of the study.	Improve recovery and reduce the time that a patient spends in the hospital	Supervised by a therapy assistant while taking part in the VR session. Assisted in wearing the headset and ensured a comfortable and correct seating position. The virtue program was started by the therapy assistant, and the difficulty of each scenario was graded depending on their needs.	Statistically significant improvement in attention and orientation No statistically significant difference in the primary outcome measures at 3 months Secondary outcomes: statistically significant reduction in anxiety scores in the mild to moderate cognitive impairment (MoCAf 15-24) treatment group
Chirico et al [38], 2020	Each chemotherapy treatment lasted for 45 to 90 minutes. Patients used the VR system for 5 to 10 minutes to become accustomed to it, and then the nurse administered the chemotherapy. In particular, participants explored an island by walking through a forest, observing different animals, climbing a mountain, and swimming in the sea.	One group of patients received music therapy, where they were provided an MPEG audio layer 3 reader and headphones 5 minutes after starting the chemotherapy infusion. Patients listened to relaxing music pretaped by an expert music therapist for 20 minutes. Another group of patients was allowed to choose common activities during treatment, such as conversation or reading.	Reduce the anxiety, depression, and fatigue related to chemotherapy through distraction	A research nurse, together with a trained psychologist, explained how to use the VR equipment and helped patients with putting on the headset.	Statistically significant reduction in anxiety levels between pretest and posttest and a significant interaction effect between the time factor and the intervention factor (time×intervention) on the patient’s level of anxiety. Furthermore, the analysis showed a significant main effect for the time factor. There was a significant reduction between the preintervention and the postintervention phases in the following negative mood states: tension, depression, anger, and fatigue (P<.001)
Groninger et al [39], 2021	The study used the Forest of Serenity (Holosphere VR), which features a 10-minute guide through a forest and waterfall with voice narration. Each assigned experience (VR or guided imagery) was administered at the bedside by the study coordinator. When the participant was ready, the coordinator left the bedside to stand immediately outside the room, started to time the experience per the protocol, and waited either for the end of the assigned experience time or for a verbal signal from the participant requesting assistance with the equipment.	The patients received a guided imagery session on a portable tablet, which had options of guided meditation, instrumental background music, and 2D imagery of a peaceful lakeside view.	VR aimed to provide distraction therapy	The study coordinator explained the equipment and positioned it, then left the room until the end or if assistance was asked for.	Statistically significant decrease in pain scores (Likert scale); the VR group had significantly lower scores than the guided imagery group (P<.001). Secondary outcomes: there was a statistically significant improvement in the total FACIT-Palg 14 score and an insignificant change in the distress score
Hsu et al [40], 2022	In addition to 20 minutes of therapist-facilitated task-specific training as a usual care session, patients each received 30 minutes of VR time. The sequences of the hand exercises of VR consisted of the movements of the forearm, supination or pronation, wrist extension or flexion, finger extension or flexion, thumb opposition with the little finger, thumb extension or flexion, and tendon-gliding exercises, which involve a series of hand movements (straight hand, hook fist, straight fist, and full fist). Each movement was repeated 50 times. The participant sat in a comfortable chair in front of a desk with the VR system.	One group of patients performed hand exercises and looked at their hand motions through a mirror. Another group of patients received OTh in addition to physical therapy and speech therapy.	Improve upper extremity movement, quality of motor movement in the affected arm, and manual dexterity	Participants received 20 minutes of therapist-facilitated task-specific training. They were supervised and assisted by the physical therapist.	Statistically significant improvement in only the FMA-UEi motor coordination (P=.002) and the MAL-AOUj (P=.002)
Huang et al [41], 2022	A total of 20 VR scenes from commercial games were selected for the participants. Selected scenes were chosen based on the original upper limb activities. Participants could accomplish the first-contact task with the help of their unaffected hand, and then, they were encouraged to use the affected hand or both hands at the same time. Upper limb movements in most scenes involved aiming, shooting, hitting, waving arms, punching, and throwing objects. Participants’ activity performance and information were recorded, including training intensity, duration, game scores, level completed, and invalid activities. This information allowed the therapist to assign the tasks, adjust the difficulty in the VR game settings, and even design a new way to play the game for each participant according to their needs and capacities.	The patients received OT.	VR aimed to improve upper extremity movement and range of motion	After the participant confirmed that the sight and sound were clear and comfortable, the controllers were handed to the participant, and the therapist could even design a new way to play the game for each participant according to their needs and capacities.	Statistically significant time effect for all items of upper limb assessment (P<.05), except FMA-UE motor coordination and speed
Huang et al [42], 2024	Participants in the imVR^k^ system received the first 30 minutes of conventional rehabilitation, and in the second 30 minutes, the rehabilitation was performed in imVR systems. Participants in the imVR group were required to complete 6 programs: frying dumplings and noodles by controlling a wok handle in a virtual kitchen; popping balloons by controlling a sword in a virtual fencing hall; punching dolls by controlling a big fist in a virtual boxing arena; playing basketball in a virtual court, in which the ball is shot by a controller and the height and distance is varied over time; collecting eggs into a virtual basket by a controller; and tidying up a desk and moving objects to a designated position in a virtual office. All participants received rehabilitation training for 5 days per week for 3 weeks.	Patients received a 60-minute conventional rehabilitation program per day.	Improve motor impairment and ADL	No training, supervision, or assistance was provided; however, in the early stages of rehabilitation, due to poor functioning of the hemiplegic side upper limb, participants used the help of their unaffected side limb.	Statistically significant improvements in upper extremity motor impairment and ADL for up to at least 12 weeks postintervention, and the magnitude of these improvements was much greater than that of other intervention programs (P=.02)
Jo et al [43], 2024	Each participant of the 360° MTG^l^ performed treatment tasks using their unaffected extremities, which were recorded with a 360° camera (Insta 360° X3; Insta 360). The patients received treatment in the same place where the videos were recorded. The therapist instructed and guided patients to use the affected side following the immersive video during all tasks. The MT^m^ tasks were the same as those of TMTG^n^ (finger flexion and extension, wrist flexion and extension, pronation and supination, and elbow flexion and extension for 10 repetitions; the tasks instructed participants to use the affected limb while following the mirror images of the unaffected limb to induce movement re-education; and playable tasks using ring toys were also provided for 5 repetitions). The 360° MT was provided for 30 minutes per session, 3 sessions a week for 4 weeks. The 360° MTG also received additional conventional physical therapy.	One group received traditional MT through an acrylic mirror that mirrored the unaffected side upper limb, where the affected side limb was hidden behind the mirror. Another group received a conventional physical therapy program consisting of warm-up, circuit, and cool-down exercises.	Upper extremity rehabilitation in patients with stroke	A therapist adjusted the device to fit the eye level of each patient. The therapist instructed and guided patients to use the affected side following the immersive video during all tasks.	FMA-UE, MFTo, and BBTp scores were significantly improved in 360° MTG and TMTG, while improvements in CGq were not significant. In group comparisons, changes in 360° MTG were significantly higher compared to changes in TMTG and CG (P<.001)
Laghlam et al [44], 2021	The VR session started during the preparative phase, at least 5 minutes before the removal of the drains, and was continued for 10 minutes after. We used a VRx helmet with a 90° field of view with head tracking. Patients had a choice between 5 different immersive environments (360° videos): a snowy mountain, a landscape in India or Camargue (France), a balloon ride, or a canoe descent.	The patients received Kalinox (Air Liquide) 1 minute before the removal of the drains. This was delivered continuously and stopped 1 minute after removal to avoid side effects.	Reduce pain during cardiac surgery, specifically during the removal of drains	The nurse supervised and assisted patients while using VR.	No statistically significant improvements in patient functional outcomes (Analgesia Nociception Index; P=.69)
Lee and Kang [45], 2020	The “Relaxing Music for Meditation” program was designed to induce meditation and consists of 8 meditation videos of 30 minutes. Scenes of mountains, seas, or lakes appear on the VR screen, and background sounds and calm music allow participants to immerse themselves in the experience. The study began on the day of admission to the ICU^r^.	The patients received a daily routine sleep intervention.	Induce meditation and improve sleep quality	No training, supervision, or assistance was mentioned; however, it took about 20 minutes for the experimental group to learn how to use the HMD.	Statistically significant increase in sleep scores (Pittsburg Sleep Quality Index), sleep time, and mean sleep efficacy in the experimental group compared to CG (P=.002)
Mekbib et al [46], 2021	The VR rehabilitation training included reaching, grasping, and releasing tasks. Following the training schema, all patients in the VR group received 1-hour VR and 1-hour OT per day, 4 days per week for 2 weeks. In each VR session, the therapy modes were set by a therapist based on the patient’s interest and actual motor capability. After choosing the therapy mode, the therapist randomly set 20 colored balls from the aerial view map. Then, the patient was instructed to reach, grasp, and release each ball into the basket. After completing the first VR session (20 balls), the therapist could set the next VR session and adjust the task complexity to be slightly higher or lower or keep it as it was in the previous session based on the patient’s activity performance.	The patients received OT.	Upper extremities to aid in the recovery of motor function after stroke and improve upper extremity function and the ability to perform ADL	The therapist helped train the patient. If the patient was not able to move their upper extremity toward the target ball, the therapist could help move the affected extremity to assist in the intended task.	Statistically significant improvement in the FMA-UE scores (P<.001) and BIs scores (P=.003)
Menekli et al [47], 2022	Patients in the intervention group were educated on the use of VR glasses by the researcher. A smartphone and the various number of parks, nature, and seaside walks; submarine; and museum tours with the Matic Music were provided to patients in the intervention group. Each of these videos took approximately 3 to 10 minutes, and patients could choose the video that they wanted to watch. Patients started to use the VR after the baseline assessment (about 1 minute before the implantation) and continued to use it until the end of the implantation. Patients were instructed to use VR after implantation when they felt pain from the implantation.	The patients received conventional care.	Pain management after implantation	Supervision and assistance were provided by a nurse, who was giving directions to patients on how to use the videos.	Statistically significant improvement in the SAI scores (P<.001) and VAS scores (P<.001)
Ögün et al [48], 2019	Patients used the VR device to play task-oriented games that focused on gripping and handling objects with arm and forearm motion and stability. A different game was used for each function, with 4 games: a cube handling game used for grip function integrated with the leap motion device to make the patients feel like they were handling a real object using their own fingers without the use of any external device to track hand motion; another leap motion–integrated game involving decorating a tree with leaves and fruits or picking up vegetables from a bowl and putting them back, which was chosen to facilitate all hand motions combined with complex motions in a task-oriented job; a kitchen experience game used for stimulating forearm supination and pronation and for combining complex arm movements; and a drumming game, selected to randomly assign each separate movement of upper extremity flexion and abduction.	The patients received conventional upper extremity active exercises comprising the same tasks as used in the VR group.	Improve upper extremity functionality and make daily activities more accessible	None mentioned	Statistically significant improvement in FMA-UE, ARATt, FIMu, and PASSv scores (P<.001)
Park et al [49], 2013	The VR-based postural control program consisted of a program for the improvement of gait ability by visual feedback compared to reference motion scenes and reality motion. The program consisted of 3 stages: trunk stability and pelvic tilting in a supine position (stage 1); trunk upright control and pelvic tilting exercise in a sitting position, and a selective movement between trunk and pelvis (stage 2); and lower extremity muscle strengthening exercise and weight bearing under maintenance of trunk stability in a standing position (stage 3). The participants were provided with specific scenes on HMD showing the simultaneous output.	The patients received targeted lower extremity muscle strengthening exercises, static and dynamic balance training, and gait training.	Improve postural control, spatiotemporal gait ability, and functional gait ability and to increase walking ability	The participant saw the prerecorded reference motion and practiced it again 3 times. No other provision of training, supervision, or assistance was mentioned.	No statistically significant improvements in patient functional outcomes
Rousseaux et al [50], 2022	A 20-minute VR session was conducted, where participants watched a 3D graphical landscape consisting of a mountain cabin near a lake at sunrise, followed by a relaxing moment in the clouds. The display was visual and audio, with sounds of ambient nature but no voice.	The patients received conventional care.	Lower anxiety and decrease pain	None mentioned	No statistically significant differences in the effectiveness, either between the techniques themselves or between the techniques and no treatment
Rutkowski et al [51], 2021	The primary aim of the software was to calm the patient down and improve his or her mood. The software features a virtual therapeutic garden and is based on the Ericksonian psychotherapy approach. The garden is a metaphor for the patient’s health: at the beginning, it appears untidy and gray; however, with each session, it becomes more colorful and alive, thus symbolizing the process of recovery of energy and vigor. The 2 groups participated in the traditional pulmonary rehabilitation program. Components were performed once a day each, for 15 to 30 minutes (depending on the task), 5 times a week for 2 weeks. Exercises were performed as follows: fitness exercises while standing on the knees and lying on the side, abdomen, and back; strengthening exercises of the diaphragm with resistance; prolonged exhalation exercise; chest percussion; inhalation with a 3% sodium chloride isotonic solution administered via an ultrasonic device; and stationary cycle ergometer exercise to obtain a training heart rate according to GOLD^w^ spirometric stages. The difference between the groups is in the type of relaxation training: the VR group performed 10 VR therapy sessions of 20 minutes, and the CG performed 10 Schultz autogenic training sessions of 20 minutes.	The patients received the traditional pulmonary rehabilitation program.	Reduce depression, anxiety, and stress levels and improve functional capacity	None mentioned	Statistically significant improvement in the HADS-Ax (P<.001), HADS-Dy (P<.001) and general HADSz (P<.001)
Shin et al [52], 2023	In the virtual world, patients were greeted by their respective attending physicians and guided through the visual and audio unfolding of the RT^aa^ process. Patients were guided virtually with 360° viewing of the different treatment environments and RT machines that they would encounter when undergoing RT. Throughout the physician’s explanation, patients were able to witness a real example of a patient with breast cancer undergoing the RT, offering patients a concrete example of what to expect on treatment days.	The patients received conventional care.	Alleviate anxiety related to undergoing RT	Patients were assisted with the proper positioning of the VR headset and noise-cancelation earphones provided by the hospital.	Significant reduction in patient anxiety immediately, not only on the primary end point, APAISab, but also on the STAIac and LASAad anxiety scales. Among the 3 anxiety scales, long-term anxiety reduction was observed only when anxiety was measured by LASA
Song and Lee [53], 2021	The VRBAT^ae^ group underwent immersive VRBAT. The VR content consisted of rehabilitation tasks to improve upper limb function, the ability to perform ADL, and visual perception. The VR tasks performed included everyday activities, such as turning on lights, organizing a chest of drawers, organizing a kitchen, watering plants, and purchasing items at a convenience store. The virtual living room, kitchen, veranda, and convenience store were designed to simulate real environments. Interventions were performed for 30 minutes a day, 5 times a week, for 4 weeks, for a total of 20 sessions. All participants in the VRBAT group also underwent an hour of conventional rehabilitation per day.	The patients performed exercises, such as turning on lights, arranging a chest of drawers, and arranging a kitchen as part of bilateral upper extremity training in a real environment.	Improve the range of motion, bilateral upper extremity movement, and upper limb function	There was some training, supervision, or assistance provided; however, this was not written, although figures in the study show someone guiding the participant.	Statistically significant improvement in the proprioception test (P<.05)
Spiegel et al [54], 2019	Participants selected one of the 21 VR experiences from an app. Patients were asked to use the headset for 10 minutes in the presence of study staff to practice with the equipment and then advised to use the headsets thrice daily, for 10 minutes per session, and later, as needed for breakthrough pain, for the subsequent 48 hours. Following these initial instructions, patients decided for themselves and in partnership with their care team whether, how frequently, and how long to use the VR equipment without direct input from study staff.	The patients watched the “Health and Wellness Channel,” which includes guided relaxation content (eg, yoga and meditation programming), discussions about health and wellness topics, and poetry readings.	Reduce pain in hospitalized patients	Participants were instructed on procedures for wearing the headset, how to select among the 21 VR experiences, and how to adjust volume and brightness. Regarding supervision, patients were initially asked to use the headset for 10 minutes with the presence and assistance of the study staff and care team.	Significant improvement in pain scores 48 hours and 72 hours postintervention using a numeric rating scale (P=.03 and P=.04, respectively)
Torres García et al [55], 2023	The sessions recreated a chemotherapy procedure. Using VR, patients managed their anxiety and fear through relaxation, attentional focus, and mindfulness strategies. The complete intervention included 3 modules, which each patient repeated during 4 individual sessions, aiming to improve their preparation and adaptation to the cancer treatment they had to start.	The patients received a regular psychoeducational intervention.	Improve patients’ anticipatory anxiety and adherence to chemotherapy and facilitate better and faster adaptation to treatment coping mechanisms	The therapist was present throughout the procedure.	Statistically significant differences in the decrease of anxiety and depression with the Anxiety and Depression Scale. At 3 months, a more notable decrease in the Emotional Discomfort Detection Scale was observed in the experimental group, and the fighting spirit and cognitive avoidance increased, which was assessed with the MINI-MACaf coping scale
Turrado et al [56], 2021	A realistic environment was generated in which the patient could experience the various steps of their admission to surgery, from the first interview with the surgeon to admission into the surgical ward, the operating room, and the postoperative recovery room. Patients completed both questionnaires to assess their basal anxiety levels. The patients in the intervention group had unlimited access to VR glasses and to the VR app. This group completed both questionnaires again on the day before the surgical procedure.	The patients received conventional care.	Reduce presurgical anxiety and depression	None mentioned	Statistically significant changes for both the HADS subscales and for those measuring status and trait in the STAI scale for patients who were exposed to the VR simulation experience (P<.001)
Uslu and Arslan [57], 2023	The patients watched and listened to beach and nature content with VR glasses for 30 minutes. The VR glasses used were Zore G04BS VR Shinecon VR glasses, compatible with smartphones (eg, Android and IOS). The contents were determined in the YouTube library and included 360° relaxing beach and nature images.	The patients received conventional care.	Reduce anxiety and fatigue levels	Patients were informed about using VR and viewing the content. From the content opened by the researcher, it was ensured that the patient watched and listened to the content of their choice.	The mean postintervention anxiety scores decreased in the intervention group. In this group, the mean posttest fatigue and subscales scores decreased in all cycles compared with the mean pretest scores
Zhang et al [58], 2023	The patients watched and listened to beach and nature content with VR glasses for 30 minutes. The VR glasses used were Zore G04BS VR Shinecon VR glasses, compatible with smartphones (eg, Android and IOS). The contents were determined in the YouTube library and included 360° relaxing beach and nature images.	The patients received conventional care.	Reducing anxiety and fatigue levels	Patients were informed about using VR and viewing the content. From the content opened by the researcher, it was ensured that the patient watched and listened to the content of their choice.	The mean postintervention anxiety scores decreased in the intervention group. In this group, the mean posttest fatigue and subscales scores decreased in all cycles compared with the mean pretest scores

^a^VR: virtual reality.

^b^VAS: visual analog scale.

^c^SAI: State Anxiety Inventory.

^d^HMD: head-mounted display.

^e^ADL: activities of daily living.

^f^MoCA: Montreal Cognitive Assessment.

^g^FACIT-Pal: Functional Assessment of Chronic Illness Therapy–Palliative Care.

^h^OT: occupational therapy.

^i^FMA-UE: Fugl-Meyer assessment for upper extremity.

^j^MAL-AOU: motor activity log-amount of use.

^k^imVR: immersive virtual reality.

^l^MTG: mirror therapy group.

^m^MT: mirror therapy.

^n^TMTG: traditional mirror therapy group.

^o^MFT: manual function test.

^p^BBT: box and block test.

^q^CG: control group.

^r^ICU: intensive care unit.

^s^BI: Barthel Index.

^t^ARAT: action research arm test.

^u^FIM: functional independence measure.

^v^PASS: performance assessment of self-care skills.

^w^GOLD: Global Initiative for Chronic Obstructive Lung Disease.

^x^HADS-A: Hospital Anxiety and Depression Scale-Anxiety.

^y^HADS-D: Hospital Anxiety and Depression Scale-Depression.

^z^HADS: Hospital Anxiety and Depression Scale.

^aa^RT: radiation therapy.

^ab^APAIS: Amsterdam Preoperative Anxiety and Information Scale.

^ac^STAI: State-Trait Anxiety Inventory.

^ad^LASA: linear analog scale assessment.

^ae^VRBAT: VR-based bilateral arm training.

^af^Mini-MAC: mini-mental adjustment to cancer.

### Participant Characteristics

In total, the studies included 1225 participants. Of the 24 included studies, 3 (12%) included patients with an average age of <50 years [43,49,58]; 8 (33%) included patients with an average age of 51 to 60 years [35,36,38-41,46,52,55,57]; 5 (21%) included patients with an average age of 61 to 65 years [42,45,48,51,52]; 3 (12%) included patients with an average age of 66 to 70 years [44,50,56]; and 1 (4%) included patients with an average age of >75 years [37]. Two studies did not report the average age of the patients, as listed in Table 2 [47,54]. No studies reported how many patients in the sample were aged >65 years. A total of 16 (67%) of the 24 included studies had a high risk of bias (Multimedia Appendix 4 [35-58]).

**Table 2:** Patient characteristics.

Study	Patients, n	Patient age (y), average (SD); range	Serious illness, n (%)	Sex, n (%)	Study location	Ethnicity
Bani Mohammad and Ahmad [35], 2018	80	52 (10.34); 30-70	Breast cancer stage: 1: 9 (11.3) 2: 32 (40) 3: 29 (36.3) 4: 10 (12.5)	Female: 80 (100)	Jordan	Information not provided
Burrai et al [36], 2023	74	59 (10.8); 33-83	Breast cancer: 35 (47.2) Colon cancer: 10 (13.5) Rectum cancer: 6 (8.1) Melanoma: 2 (2.7) Non-Hodgkin lymphoma: 1 (1.4) Stomach cancer: 2 (2.7) Ovary cancer: 5 (6.8) Pancreas cancer: 4 (5.4) Uterine cervix cancer: 1 (1.4) Larynx cancer: 2 (2.7) Esophagus cancer: 1 (1.4) Testicle cancer: 1 (1.4) Prostate cancer: 1 (1.4) Lung cancer: 2 (2.7) Biliary tract cancer: 1 (1.4)	Female: 55 (74.3) Male: 19 (25.7)	Italy	Italian: 24 (97.2) Not Italian: 1 (0.8)
Chatterjee et al [37], 2022	40	Average age: not reported (SD 17.67) Intervention median (IQR) age: 77.5 (43-89) years Control median (IQR) age: 63 (29-86) years	Ischemic: 34 (85) Hemorrhagic: 6 (15)	Female: 19 (47.5) Male: 21 (52.5)	United Kingdom	Information not provided
Chirico et al [38], 2020	92	55 (6.00)	Breast cancer stages: 1: 24 (26.1) 2: 39 (42.4) 3: 29 (31.5)	Female: 92 (100) Male: 0 (0)	Italy	Information not provided
Groninger et al [39], 2021	88	56 (13.24)	Heart failure: 19 (51.4) Other cardiac diseases: 5 (13.5) Other medical and surgical problems: 13 (35.1)	Female: 35 (39.8) Male: 53 (60.2)	United States	African American: 74 (84.1) Hispanic: 1 (1.1) White: 13 (14.8)
Hsu et al [40], 2022	52	55 (12.11)	Stroke type: Ischemic: 29 (55.8) Hemorrhagic: 23 (44.2)	Female: 32 (61.5) Male: 20 (38.5)	Taiwan	Information not provided
Huang et al [41], 2022	30	58 (11.78) Intervention group range: 22-70 Control group range: 28-71	Infarction: 22 (73) Hemorrhage: 8 (27)	Female: 20 (67) Male: 10 (33)	Taiwan	Information not provided
Huang et al [42], 2024	40	64 (10.99)	Stroke type: Ischemic: 37 (92.5) Hemorrhagic: 3 (7.5)	Female: 16 (40) Male: 24 (60)	China	Information not provided
Jo et al [43], 2024	45	50 (13.36)	Stroke paretic side: Right: 23 (51) Left: 22 (49)	Female: 22 (49) Male: 23 (51)	South Korea	Information not provided
Laghlam et al [44], 2021	180	68 Intervention group range: 61.5-75.0 Control group range: 60.0-74.0	Isolated coronary bypass surgery: 99 (55) Combined coronary bypass surgery: 7 (3.9) Isolated or combined valve surgery: 61 (33.9) Aorta surgery: 5 (2.8) Myxoma: 1 (0.6) Pericardiocentesis for tamponade: 6 (3.3) Abdominal aorta surgery: 1 (0.6)	Female: 46 (25.56) Male: 134 (74.4)	France	Information not provided
Lee and Kang [45], 2020	48	66 (16.40)	CHFa: 6 (12.5) Cardiomyopathy: 2 (4.2) MIb: 26 (54.2) Others: 12 (25.1)	Female: 16 (3.33) Male: 32 (66.7)	South Korea	Information not provided
Mekbib et al [46], 2021	23	56 (10.96)	Stroke type: Ischemic: 17 (73.9) Hemorrhagic: 6 (26.1)	Female: 6 (26.1) Male: 17 (73.9)	China	Information not provided
Menekli et al [47], 2022	139	Average age: not reported Age by category (years), n (%): 20-30: 23 (16.55); 31-40: 34 (24.46); 42-52: 60 (43.17); 53-63: 22 (15.83)	Breast cancer: 53 (38.1) Lung cancer: 46 (33.1) Stomach (gastric) cancer: 18 (12.9) Colon cancer: 12 (8.6) Pancreas cancer: 10 (7.2)	Female: 87 (62.6) Male: 52 (37.4)	Turkey	Information not provided
Ögün et al [48], 2019	65	61 (9.62)	Stroke: 65 (100)	Female: 14 (22) Male: 51 (78)	Turkey	Information not provided
Park et al [49], 2013	16	48 (7.89)	Stroke type: Ischemic: 9 (56.3) Hemorrhagic: 7 (43.7)	Female: 5 (31.25) Male: 11 (68.75)	Korea	Information not provided
Rousseaux et al [50], 2022	100	66 (11.50)	Coronary artery bypass graft surgery: 57 (57) Mitral valve replacement: 26 (26) Multiple interventions: 4 (4) Others: 4 (4)	Female: 24 (24) Male: 76 (76)	Belgium	Information not provided
Rutkowski et al [51], 2021	50	64 (7.77)	COPDc: 50 (100)	Female: 41 (82) Male: 9 (18)	Poland	Information not provided
Shin et al [52], 2023	196	47.5 (7.63)	Breast cancer stages: Stage 0: control group: 28 (28.3); VR group: 15 (15.5) Stage 1A: control group: 43 (43.4); VR group: 47 (48.5) Stage 1B: control group: 3 (3); VR group: 8 (8.3) Stage 2A: control group: 16 (16.2); VR group: 21 (21.7) Stage 2B: control group: 5 (5.1); VR group: 2 (2.1) Stage 3A: control group: 2 (2); VR group: 2 (2.1) Stage 3C: control group: 1 (1); VR group: 1 (1Stage 4: control group: 1 (1); VR group: 1 (1)	Female: 196 (100)	South Korea	Information not provided
Song and Lee [53], 2021	10	64 (9.18) Intervention group range: 51-80 Control group range: 51-80	Hemorrhagic stroke: 3 (30) Ischemic stroke: 7 (70)	Female: 4 (40) Male: 6 (60)	Korea	Information not provided
Spiegel et al [54], 2019	120	60 (15.50)	Intervention: Gastrointestinal: 13 (10.8) Infectious disease: 15 (12.5) Internal medicine: 25 (20.8) Oncology: 10 (8.3) Orthopedics: 36 (30) Other: 21 (17.5)	Female: 60 (50) Male: 60 (50)	United States	African American: 21 (34.4) White: 38 (62.3) Other: 2 (3.3)
Torres García et al, 2023 [55]	133	49 (11.6); 30-82	Breast cancer: 133 (100)	Female: 133 (100)	Spain	Information not provided
Turrado et al [56], 2021	126	64 Intervention group range: 41-85 Control group range: 50-86	Colorectal cancer: 58 (46) Right colectomy: 21 (16.7) Sigmoidectomy: 20 (15.9) LARd+TaTMEe: 25 (19.8) Transverse colon resection: 2 (1.6)	Female: 53 (42) Male: 73 (58)	Spain	Information not provided
Uslu and Arslan [57], 2023	66	52 (8.89)	Breast cancer patients receiving adjuvant chemotherapy: 66 (100)	Female: 66 (100)	Turkey	Information not provided
Zhang et al [58], 2023	60	34.38 (10.76)	Acute myeloid leukemia: 47 (78.33) Lymphoblastic leukemia: 1 (21.67)	Female: 33 (55) Male: 27 (45)	China	Information not provided

^a^CHF: congestive heart failure.

^b^MI: myocardial infarction.

^c^COPD: chronic obstructive pulmonary disease.

^d^LAR: low anterior resection.

^e^TaTME: transanal total mesorectal resection.

### Serious Illness

Stroke, cancer, and cardiovascular disease were the most common types of serious illnesses for which VR interventions were used to impact the outcomes related to patients’ quality of life. Out of 24 included studies, 9 (38%) focused on patients who experienced stroke, which included both ischemic stroke and hemorrhagic stroke [37,40-43,46,48,49,53], and 9 (38%) included patients who were diagnosed with some type of cancer, varying in the stages of cancer in each patient [35,36,38,42,43,47,52,55-58]. In 5 (21%) of the 24 studies, only patients who were diagnosed with breast cancer were included; 1 study (4%) included patients with colorectal cancer, and 1 (4%) included patients with heterogeneous types of cancer. Four (17%) out of 24 studies included patients who had some form of cardiovascular disease, including heart failure and other conditions that required cardiac surgery [39,44,45,50]. One study included patients with chronic obstructive pulmonary disease (COPD), and another included patients who reported pain during their hospitalization [51,54].

### Purpose and Content of VR

In 9 (38%) out of 24 included studies, the main purpose of VR was to improve mobility and strength in the patient, ranging from upper extremity, lower extremity, and pelvic or abdominal region to a combination of these regions [37,40-43,46,48,49,53]. All of these mobility studies also included patients who experienced strokes. The VR media used in these studies encouraged active participation from the patients in various tasks and games. Some examples of VR media used to help improve mobility and strength in patients’ poststroke treatment were daily exercises that encouraged increased range of motion, strength for specific movements, and reach and grasp through the use of balls. A total of 8 (89%) of the 9 studies that used VR for the purpose of improving mobility and strength improved patient quality of life–related outcomes [37,40-43,46,48,53].

There was some overlap in the protocols and diseases of the additional studies. Out of 24 studies, 12 (50%) used VR distraction therapy to reduce pain in patients with cancer or, cardiovascular disease or hospitalized patients [35,36,38,39,42-44,47,50,52,54-58]. A total of 9 (38%) out of 24 studies used VR to reduce anxiety in patients with cancer, cardiovascular disease, or COPD [36,38,42,43,50-52,55-58]. In studies aiming to reduce pain and anxiety, patients experienced immersive natural scenery and comforting music while comfortably sitting or lying down. In another study, to reduce anxiety, patients experienced the hospital environment and could visualize and review all the steps of an upcoming procedure [56]. In another study, patients with cardiovascular disease went through a visual guided meditation track with the VR media for patients with cardiovascular disease [45].

### Training, Supervision, and Assistance

In 18 (75%) out of 24 studies, staff or physical therapists either trained patients on VR use before the intervention or supervised or assisted them through the experiment. Supervision was defined as the research team watching over the participants, ensuring their use and adherence to study guidelines. However, assistance was defined as the research team supporting patients during the intervention without constant watching throughout the experiment. A total of 5 (20.8%) out of 24 studies included VR training, supervision, and assistance, all of which reported improvements in patient quality of life–related outcomes [38,40,43,46,54]. Of 24 studies, 7 (29.2%) included VR supervision and assistance only [36,37,41,44,47,53,57], and 2 (8.3%) included VR training and assistance only [39,49]. Two (8.3%) out of 24 studies included only VR supervision while patients were using VR [35,55]. In studies that included a variation of personnel working with the patient, only 1 staff member physically accompanied the patient in the room, where they freely communicated with the patient to fulfill their role. During a typical training, the staff member would walk the patient through the VR activity and teach them how to adjust the headset. Out of 24 studies, 6 (25%) did not describe training, supervision, or assistance with the use of VR [42,45,48,50,51,56].

### Frequency and Duration of Intervention on Quality of Life Outcomes

The frequency and duration of VR experience varied based on the intervention purpose; interventions aiming to improve pain or anxiety were shorter. while those aiming to improve mobility and strength were longer. Out of the 24 included studies, 9 (38%) studies with pain or anxiety reduction as a goal, involved only 1 VR session, ranging from 8 minutes to 4 hours (ie, patients could use VR at any time within a 4-hour window) [35,38,39,44,47,50,52,56]. A total of 7 (78%) of these 9 studies had improvements in pain and anxiety metrics, such as the Hospital Anxiety and Depression Scale (HADS), State Anxiety Index, or the Numeric Pain Scale (NPS). One study, which conducted a single 30-minute meditation session, showed improvement in sleep scores of patients with cardiovascular disease [45]. In 1 case where VR was used for 10 minutes per session, for 3 sessions per day for 2 days, patients experienced statistically significant improvement in the NPS (*P*<.04) [54]. Patients in another study who used VR for 20 minutes per session for 5 sessions per week for 2 weeks had statistically significant improvement in the HADS (*P*<.001) [51].

In the 9 studies aimed at improving mobility and strength in patients who had a stroke, VR was used for a longer duration than in pain and anxiety studies. Out of 9 studies, 6 (67%) lasted between 2 and 4 weeks [37,42,43,46,49,53], while 3 (33%) lasted between 5 and 9 weeks [40,41,48]. A total of 8 (89%) of these 9 studies showed an improvement in the patient’s functional capacity measured via the Fugl-Meyer upper extremity assessment, proprioception, or orientation metrics.

### Gold Standard Adherence

As shown in Table 3, 7 studies adhered to all the 8 gold standard VR consensus standards criteria (100%) [41,43,44,48,51,52,54], 10 adhered to 7 criteria (88%) [36,37,39,40,42,46,55-58], 6 adhered to 6 criteria (75%) [35,38,47,49,50,53], and 1 adhered to 5 criteria (62%). The reasons for not adhering to the criteria were inadequate blinding, concealment of allocation, reporting of the trial, and a priori failure to justify study duration. The studies that did not report the trial via World Health Organization International Clinical Trials or Clinicaltrials.gov originated from countries where it may not be standard practice to report the clinical trial before the study initiation. All 24 assessed studies received the institutional review board or ethics committee approval. A total of 4 (17%) of the 24 included articles were published before the 2019 study by Birckhead et al [22] that defined the gold standard VR consensus standards criteria.

**Table 3:** Study adherence to the “gold standard.”

Study		Patient population	Clinical setting	Control and randomization	Blinding and concealment of allocation	End points	Study duration	Presentation and analysis of results	Reporting the trial
**Bani Mohammad and Ahmad [35], 2019**									
	Met criteria	Completely met	Completely met	Completely met	Not completely met	Completely met	Completely met	Completely met	Not completely met
	Description	A total of 80 patients (average age of 52 years) with breast cancer	Medical and surgical wards of a specialized cancer center in Jordan	Random assignment was done based on flipping a coin; if heads appeared, then the first participant was placed in the intervention group. The rest of the participants were placed in the study groups by the order of meeting the eligibility criteria.	N/A^a^	The VAS^b^ was used to measure pain, and the SAI^c^ was used to measure anxiety.	One session occurred for 20 to 30 minutes; VR^d^ was timed with peak morphine efficacy and the goal was acute pain relief.	There was a statistically significant improvement in pain scores and anxiety levels, and no MCIDe was mentioned.	None mentioned outside of ethics or IRB^f^ approvals from individual hospitals

**Burrai et al [36], 2023**									
	Met criteria	Completely met	Completely met	Completely met	Not completely met	Completely met	Completely met	Completely met	Completely met
	Description	A total of 74 patients (average age of 59 years) with cancer	Regional hospital in Italy	An independent researcher used a random number generation software with simple randomization. A total of 3 numerical codes for the 3 allocation groups were used: 1 for the VR group, 2 for the narrative medicine group, and 3 for the standard care group. To implement the random allocation sequence, sequentially numbered, opaque, sealed envelopes were used.	Owing to the nature of VR and narrative medicine interventions, it was impossible to obtain a blinding between the participants and knowledge of the group, so this study is open-label type.	Anxiety was measured with the STAI-Y1^g^; fatigue was measured with the revised Piper Fatigue Scale; and pain was measured with the VAS scored from 0 to 10, where 0 indicates no pain and 10 indicates the worst possible pain.	VR was administered just after AT^h^ began and the immersion duration was 30 minutes.	Mean anxiety significantly decreased over time for the VR group and narrative medicine group. There was no significant difference in levels of fatigue, but overall levels of fatigue decreased for participants in the VR intervention. There was no significant difference in pain levels.	The study was registered in the US National Institutes of Health (NCT05629507).
**Chatterjee et al [37], 2022**									
	Met criteria	Completely met	Completely met	Completely met	Not completely met	Completely met	Completely met	Completely met	Completely met
	Description	A total of 40 patients (average age: not provided, median 77.5 years) with unilateral stroke	Stroke unit at the Countess of Chester Hospital NHS^i^ Foundation Trust.	Participants were randomized on a 3:1 allocation basis.	This study was not effective to implement as a double-blind trial, as most patients were able to deduce whether they were a part of the treatment group.	The MoCA^j^was the primary measure used and the NEADL^k^, HADS^l^, and quality of life (EuroQoL^m^) were the secondary measures used.	A total of 5 days a week for up to 2 weeks before their hospital discharge time that each patient spent in a VR session was very specific to the individual. Time spent in a session would depend on their level of wellness on the day and how tired they were feeling.	There was a statistically significant improvement in attention and orientation. There were no statistically significant differences in the primary outcome measures at 3 months. Secondary outcomes: there was a statistically significant reduction in anxiety scores in the mild to moderate cognitive impairment (MoCA 15-24) treatment group.	The trial was registered in the ISRCTN^n^ registry (ISRCTN16608742).
**Chirico et al [38], 2020**									
	Met criteria	Completely met	Completely met	Completely met	Not completely met	Completely met	Completely met	Completely met	Not completely met
	Description	A total of 58 patients (average age of 55 years) with breast cancer Tumor stages, n (%): 1: 8 (26.6); 2: 13 (43.3); 3: 9 (30)	National Cancer Institute, IRCCS “Fonda-zione G. Pascale,” Naples, Italy	Patients were randomly assigned to the VR or music therapy group and were compared with a nonconcurrently recruited CG^o^.	Participants and personnel knew which groups were control and intervention groups.	SAI for adults and SV‐POMS^p^	Each chemotherapy treatment lasted for 45 to 90 minutes, but the patient used VR for 20 minutes.	There was a statistically significant reduction in anxiety levels between the pretest and posttest and a significant interaction effect between the time factor and the intervention factor (time ×intervention) on the patient’s level of anxiety. Furthermore, the analysis showed a significant main effect for the time factor and significant reduction between the preintervention and the postintervention phases in the following negative mood states: tension, depression, anger, and fatigue.	None mentioned outside of ethics or IRB approvals from individual hospitals.
**Groninger et al [39], 2021**									
	Met criteria	Completely met	Completely met	Completely met	Not completely met	Completely met	Completely met	Completely met	Completely met
	Description	A total of 88 patients (average age of 56 years) with ACC or AHAq stage C or D heart failure	A 912-bed academic hospital located within MWHC^r^ in Washington, DC.	Participants were randomized by the study coordinator on a 1:1 basis using a computerized randomized scheme.	The nature of the study prevented participants and the study coordinator from being blinded to assigned interventions.	Self-reported pain scores and the FACIT-Pal 14^s^ item scale were used.	There was 1 session for 10 minutes. In distraction therapy research, there is currently no predetermined time threshold for the effect on pain experience; 10 minutes falls within the range of time frames (2 to 15 minutes) that have demonstrated the benefit of using VR for pain management.	There was a statistically significant improvement in pain score.	The trial was registered on ClinicalTrials.gov (identifier: NCT04572425).
**Hsu et al [40], 2022**									
	Met criteria	Completely met	Completely met	Completely met	Completely met	Completely met	Completely met	Completely met	Not completely met
	Description	A total of 52 patients (average age of 55 years) with stroke	Department of physical medicine and rehabilitation at a medical center in South Taiwan	Following eligibility screening, patients meeting the inclusion criteria were randomly allocated to conditions using opaque envelopes with computer-generated random numbers that the investigator opened upon receiving a consenting participant.	Eligible patients were randomly allocated until all the available envelopes had been exhausted, resulting in a 1:1:1 ratio in the MT^t^, COT^u^, or VR-MT group.	The FMA-UE^v^ motor coordination score and the MAL^w^ were used.	The study duration was 30 minutes twice a week for 9 weeks. VR timing was time-matched to the control group.	There was a statistically significant improvement in only the FMA-UE motor coordination and the MAL amount of use; no MCID was mentioned.	None mentioned outside of ethics or IRB approvals from individual hospitals
**Huang et al [41], 2022**									
	Met criteria	Completely met	Completely met	Completely met	Completely met	Completely met	Completely met	Completely met	Completely met
	Description	A total of 30 patients (average age of 58 years) with stroke infarction hemorrhage and ischemic stroke	The VR equipment was installed in a room without external disturbances, and the virtual environment was set in a 6 m^2^ physical space in a hospital in southern Taiwan.	A total of 30 patients with chronic stroke were randomized to the VR or COT groups	Clinical assessments were performed within 1 week before and after the interventions by another therapist not involved in training and blinded to the purpose and group allocation.	The FMA-UE and AROM^x^ were used.	All participants received 16 sessions of intervention for 60 min per day, 2 to 3 days per week.	There was a statistically significant time effect for all items of upper limb assessment except FMA-UE motor coordination and speed.	The trial was registered in the WHO^y^ International Clinical Trials registry (ChiCTR2100047853).

**Huang et al [42], 2024**									
	Met criteria	Completely met	Completely met	Completely met	Not completely met	Completely met	Completely met	Completely met	Completely met
	Description	A total of 40 patients (average age of 63 years) with ischemic or hemorrhagic stroke	Second Affiliated Hospital and Yuying Children’s Hospital of Wenzhou Medical University, China	Each participant was randomly assigned a code based on computer-generated, permuted block randomization with a block size of 4.	Because of the nature of the intervention, participants and therapists could not be blinded to the allocated treatment. These therapists did not participate in assessments of the outcomes.	The FMA-UE [20] and the BI^z^ were used.	All participants received rehabilitation training for 5 days per week for 3 weeks. The first 30 minutes was conventional rehabilitation, followed by 30 minutes of rehabilitation performed in imVR^aa^ systems	There were statistically significant improvements in FMA-UE motor impairment and BI scores.	The trial was registered on ClinicalTrials.gov (identifier: NCT03086889).
**Jo et al [43], 2024**									
	Met criteria	Completely met	Completely met	Completely met	Completely met	Completely met	Completely met	Completely met	Completely met
	Description	A total of 45 patients (average age of 50 years) with stroke	Hospital in Seoul, Republic of Korea	Randomized controlled trial with the participants randomly allocated into 3 groups using a computer-generated list of numbers at a 1:1:1 ratio: imVR-based 360° MTG^ab^, TMTG^ac^, and a CG	The study was conducted as an assessor-blinded and randomized controlled trial.	FMA-UE was used, and secondary outcome measurements were MFT^a^^d^ and BBT^a^^e^.	In addition to conventional physical therapy, traditional MT was provided for 30 minutes per session, 3 sessions a week, for 4 weeks	There was a significant improvement in FMA-UE, MFT, and BBT in 360° MTG and TMTG and changes in 360° MTG were significantly higher compared to TMTG and CG.	The trial was registered on ClinicalTrials.gov (NCT05796843) and received approval from the Sahmyook University IRB (SYU 2023-01-009-001)

**Laghlam et al [44], 2021**									
	Met criteria	Completely met	Completely met	Completely met	Completely met	Completely met	Completely met	Completely met	Completely met
	Description	A total of 180 patients (average age of 68 years) with cardiac surgery	ICU^af^ of the Centre Médico-Chirurgical Ambroise Paré in Neuilly-sur-Seine, France.	Randomization was performed using an external interactive web response system.	Patients were randomly assigned (1:1) in permuted blocks.	The NRSs^ag^ for pain and anxiety were used.	One session started at least 5 minutes before the removal of the drains and continued for 10 minutes after the removal of the drains. VR was timed with morphine administration (and the goal was for acute pain relief).	There were no statistically significant improvements in patient functional outcomes; no MCID was mentioned.	The trial was registered on ClinicalTrials.gov (NCT03956264).
**Lee and Kang [45], 2020**									
	Met criteria	Completely met	Completely met	Completely met	Not completely met	Completely met	Did not completely meet	Completely met	Not completely met
	Description	A total of 48 patients (average age of 63 years) with cardiovascular disease	Cardiac ICU, Dong-A University Medical Center, Busan, South Korea.	Patients were randomly allocated with a 1:1 random list order using the random allocation software program (version 2.0.0). ICU nurses did not know which group the next participant would be assigned to.	Double blindness was not possible due to the interventional characteristics of the study using VR equipment.	The PSQI^a^^h^ was used.	The study duration was 30 minutes before bedtime (9 PM to 11 PM) on the day of ICU admission. The difference in sleep quality and the effects of intervention during the ICU stay could not be investigated	There was a statistically significant higher PTSI score in the VR group.	Nothing was mentioned other than the IRB approval.
**Mekbib et al [46], 2021**									
	Met criteria	Completely met	Completely met	Completely met	Completely met	Completely met	Completely met	Completely met	Not completely met
	Description	A total of 23 patients (average age of 56 years) with stroke	Department of Rehabilitation Medicine at Zhejiang Province People’s Hospital (Hangzhou, China).	This clinical trial used a single-blind, randomized, parallel group design. Patients were randomly assigned to either the VR or OT^a^^i^ group using random numbers generated by a computer program.	The group allocation procedure was managed by a physician who was unaware of the study protocol.	The FMA-UE and BI scores were used.	Study duration was 1 hour VR and 1 hour OT per day, 4 days per week for 2 weeks. VR timing was time-matched to the CG.	There was a statistically significant improvement in FMA-UE and BI scores; no MCID was mentioned.	None mentioned outside of ethics or IRB approvals from individual hospitals and the Helsinki Declaration of Ethical Principles for Medical Research Involving Human Subjects

**Menekli et al [47], 2022**									
	Met criteria	Completely met	Completely met	Completely met	Not completely met	Completely met	Not completely met	Completely met	Completely met
	Description	A total of 139 patients (average age: not provided) with cancer	Malatya Turgut Özal University Oncology Hospital	Computer-assisted simple randomization was used to determine the groups.	There was no blinding for the patients or the researchers throughout the study.	The SAI and VAS scores were used.	The study was conducted at the liberty of the patient throughout the procedure, lasting 4 hours.	There was a statistically significant improvement in the SAI and VAS scores; no MCID was mentioned.	The trial was registered on ClinicalTrials.gov (NCT05140707).

**Ögün et al [48], 2019**									
	Met criteria	Completely met	Completely met	Completely met	Completely met	Completely met	Completely met	Completely met	Completely met
	Description	A total of 65 patients (average age of 61 years) with ischemic stroke	Bolu Abant Izzet Baysal University, Physical Therapy and Rehabilitation Hospital	Patients were randomly divided into 2 groups, VR or control, with stratified randomization according to age, sex, and stroke onset, using an online randomization website.	Both patients and outcome assessors were masked, achieved using sham VR therapy with the CG, with the outcome assessor blinded to the groups.	FMA-UE, ARAT^aj^, FIM^ak^, PASS-IADL^al^, and PASS-BADL^am^ were used as secondary outcome measurements.	The VR group received VR rehabilitation 3 days a week, on Monday, Wednesday, and Friday, at the same time each day for 6 weeks. Each session lasted approximately 60 minutes and comprised 4 games that lasted 15 minutes each.	There was a statistically significant improvement in FMA-UE, ARAT, FIM, and PASS scores.	The trial was registered on ClinicalTrials.gov (NCT03135418).

**Park et al [49], 2013**									
	Met criteria	Completely met	Completely met	Completely met	Not completely met	Completely met	Completely met	Completely met	Not completely met
	Description	A total of 16 patients (average age of 48 years) with stroke	Stroke units of a hospital in Seoul, Korea.	The 16 participants were randomly assigned to either the experimental group (n=8) or the CG (n=8) by selection of white or black go stones 1 hour before the start of the pretest.	N/A	The patient’s spatiotemporal gait ability, functional gait ability, and increased functional walking ability were measured.	The study duration was 60 minutes per day, 5 days per week for 4 weeks. VR timing was time-matched to the CG.	There were no statistically significant improvements in patient functional outcomes; no MCID was mentioned.	None mentioned outside of ethics or IRB approvals from individual hospitals

**Rousseaux et al [50], 2022**									
	Met criteria	Completely met	Completely met	Completely met	Not completely met	Completely met	Not completely met	Completely met	Completely met
	Description	A total of 100 patients (average age of 66 years) who underwent cardiac surgery	Liege University Hospitals, Domaine Universitaire du Sart Tilman	Randomization was undertaken in blocks of 5 patients.	Given the nature of the techniques, neither the patients nor the investigators were blinded to the treatment assignment.	The VAS was used.	The study comprised a 20-minute session on the preoperative day (T0 and T1) and a 20-minute session after surgery (T2 and T3). No justification was written in the manuscript for the study duration.	There was no statistically significant difference in effectiveness between hypnosis and VR	The trial was registered retrospectively on ClinicalTrials.gov (NCT03820700).
**Rutkowski et al [51], 2021**									
	Met criteria	Completely met	Completely met	Completely met	Completely met	Completely met	Completely met	Completely met	Completely met
	Description	A total of 50 patients (average age of 64 years) with COPDan	A specialist Hospital in Głuchołazy, Poland	Randomization was performed using the Research Randomizer (ratio of 1:1), a web-based service that offers instant random assignment. Sealed envelopes were used for group assignments.	An assessor-blinded parallel group	The HADS, PSQ^ao^, 6MWT^a^^p^, and lung function test with FEV1^aq^ were used.	The study was conducted once a day, each session for 15 to 30 minutes (depending on the task), 5 times a week for 2 weeks. VR group performed 10 VR therapy sessions of 20 minutes.	There was statistically significant improvement in the HADS-Aar, HADS-Das, and general HADS.	The trial was registered on ClinicalTrials.gov (NCT0460154).
**Shin et al [52], 2023**									
	Met criteria	Completely met	Completely met	Completely met	Completely met	Completely met	Completely met	Completely met	Completely met
	Description	A total of 196 patients (average age of 47.5 years) with breast cancer	Academic hospital in Seoul, South Korea.	Randomization was done using an online randomizing tool (www.randomizer. org) by an independent research coordinator.	Physicians were blinded to the study arms and did not interact with the coordinator.	Anxiety levels were measured using the APAIS^at^ as the primary end point and the STAI and LASA^au^ as secondary end points	The study was for a single time; on the same day of randomization, patients watched the 7- to 8-minute-long VR video.	There was a statistically significant reduction in patient anxiety immediately and on the primary end point, APAIS, and on the STAI and LASA anxiety scales. Long-term anxiety reduction was observed only when anxiety was measured by LASA.	The protocol was registered on ClinicalTrials.gov (NCT04141943) and approved by the Severance Hospital IRB (4-2019-0795).
**Song and Lee [53], 2021**									
	Met criteria	Completely met	Completely met	Completely met	Not completely met	Completely met	Completely met	Completely met	Not completely met
	Description	A total of 10 patients (average age of 64 years) with stroke with chronic hemiplegia	C Rehabilitation Hospital or G Rehabilitation Hospital in Gwangju city	Randomization was achieved using the opaque sealed envelope method.	N/A	Manual function test and proprioception test	Interventions were performed for 30 minutes a day, 5 times a week, for 4 weeks, for 20 sessions.	There was a statistically significant improvement in the proprioception test.	None mentioned outside of ethics or IRB approvals from individual hospitals

**Spiegel et al [54], 2019**									
	Met criteria	Completely met	Completely met	Completely met	Completely met	Completely met	Completely met	Completely met	Completely met
	Description	A total of 120 patients (average age: not provided) with pain	Cedars-Sinai Medical Center, a large, urban, tertiary care hospital	Patients were randomized on a 1:1 basis between groups using the Microsoft Excel random number generator.	A script that used neutral language regarding both interventions was used. Investigator interactions with the study participant were minimized, relying on nonstudy nursing staff to collect pain scores.	An NRS of patient-reported pain was used.	The study duration was 10 minutes per session, 3 times a day, for 48 hours; 10 minutes was selected to reduce the risk of developing cybersickness. Longer exposure times are associated with a higher risk of cybersickness.	There was a statistically significant improvement in pain scores 48 hours and 72 hours postintervention.	The trial was registered on ClincialTrials.gov (NCT02887989).

**Torres García et al [55], 2023**									
	Met criteria	Completely met	Completely met	Completely met	Not completely met	Completely met	Completely met	Completely met	Completely met
	Description	A total of 133 patients (average age of 49 years) with breast cancer	OCCU^av^ of the CIMA^aw^ Campus, Milenium Iradier Medical Center, Barcelona, Spain	Randomization was carried out using a random number list obtained with the Random.org app. The order in which it was generated was unknown to the oncologist.	The oncologist was unaware of the information provided other than the order in which randomization was generated.	HADS, DDE^ax^ scale, and the Mini-MAC^ay^ coping scale were used.	The study comprised 4 sessions lasting approximately 30 to 45 minutes. The first 3 sessions coincided with the chemotherapy treatment prescribed by the oncologist. The fourth session was conducted 3 months after the third session to check whether the benefits of the previous interventions were maintained.	There was a statistically significant reduction in anxiety and depression and significant improvements in emotional distress and coping mechanisms, including fighting spirit, cognitive avoidance, and anxious worry.	The study was approved by the Clinical Research Ethics Committee (CEIC) of Hospital CIMA (Barcelona, Spain), a center authorized by the Department of Health (H08621946).

**Turrado et al [56], 2021**									
	Met criteria	Completely met	Completely met	Completely met	Not completely met	Completely met	Completely met	Completely met	Completely met
	Description	A total of 126 patients (average age of 66 years) with the following condition, n (%): colorectal cancer: 58 (46); right colectomy: 21 (16.7); sigmoidectomy: 20 (15.9); LARaz+TaTMEba: 25 (19.8); transverse colon resection: 2 (1.6)	Third-level Academic Center in the Gastrointestinal Surgery Department in Barcelona, Spain	Patients were randomized using en bloc randomization with random block sizes.	Patients and health care professionals could not be blinded regarding group assignment.	HAD-D, HAD-A, STAI_A/S, and STAI_A/T scores were used.	The patients in the intervention group had unlimited access to VR glasses and to the VR app.	There was a statistically significant improvement for both the HADS-A and HADS-D subscales and those measuring status and trait in STAI scales in patients who were exposed to the VR simulation experience.	The trial was registered on ClinicalTrials.gov (NCT04058600).
**Uslu and Arslan [57], 2023**									
	Met criteria	Completely met	Completely met	Completely met	Not completely met	Completely met	Completely met	Completely met	Completely met
	Description	A total of 66 patients (average age of 52 years) with breast cancer	Oncology outpatient department of a university hospital in Turkey	This study was conducted using the randomization block method (6:5). Combinations with randomization were enumerated using a web-based random queue generator with permuted random block allocation (block size: 33). The intervention group and CG were determined by lots.	Blinding was not possible due to the nature of the study. With VR, the patients could not be blinded during the intervention because they knew that they were in the intervention group. Randomization was prepared by a statistician, but this was hidden from the researcher performing the study.	The STAI scale and the CFS^bb^ were used.	The study was conducted once every 21 days for 4 sessions (1 session per chemotherapy cycle for 4 cycles) for 30 minutes.	There was a statistically significant decrease in mean postintervention anxiety scores and mean posttest fatigue.	The trial was registered with the US National Institutes of Health Clinical Trials Registry on September 12, 2021 (NCT05168696)
**Zhang et al [58], 2023**									
	Met criteria	Completely met	Completely met	Completely met	Not completely met	Completely met	Completely met	Completely met	Completely met
	Description	A total of 60 patients (average age of 34 years) with leukemia	A public hospital in Guangzhou, China	Participants were randomly assigned to the intervention group and CG in a 1:1 ratio by generating 70 codes written on identically sized pieces of paper, which were placed in opaque, sealed, and sequentially numbered envelopes.	Owing to the nature of VR intervention, blinding the participants and the interventionist was impossible.	The SAI scale, CES-D^bc^ scale, and FACT-leu^bd^ questionnaire were used.	From days 1 to 14, participants experienced 14 different immersive videos. To avoid interruption from common treatment activities, every intervention occurred from 3 PM to 6 PM.	Those in the intervention group demonstrated a significantly greater reduction in anxiety (*P*<.05) and improvement in quality of life (*P*=.04). There was no significant difference in depression levels between groups (*P*=.09), although a decreasing trend was observed in the intervention group.	The protocol was registered retrospectively in the ISRCTN registry (ISRCTN 84842464; June 6, 2022).

^a^N/A: not applicable.

^b^VAS: Visual Analog Scale.

^c^SAI: State Anxiety Inventory.

^d^VR: virtual reality.

^e^MCID: minimal clinically important difference.

^f^IRB: institutional review board.

^g^STAI-Y1: State-Trait Anxiety Inventory-Y1.

^h^AT: antiblastic therapy.

^i^NHS: National Health Service.

^j^MoCA: Montreal Cognitive Assessment.

^k^NEADL: Nottingham extended activities of daily living.

^l^HADS: Hospital Anxiety and Depression Scale.

^m^EuroQOL: European Quality of Life.

^n^ISRCTN: International Standard Randomized Controlled Trial Number.

^o^CG: control group.

^p^SV-POMS: short version of profile of mood states.

^q^AHA or ACC: American Heart Association or American College of Cardiology.

^r^MWHC: MedStar Washington Hospital Center.

^s^FACIT-Pal 14: Functional Assessment in Chronic Illness Therapy–Palliative Care 14-item scale.

^t^MT: mirror therapy.

^u^COT: conventional occupational therapy.

^v^FMA-UE: Fugl-Meyer assessment for upper extremity.

^w^MAL: motor activity log.

^x^AROM: active range of motion.

^y^WHO: World Health Organization.

^z^BI: Barthel Index.

^aa^imVR: immersive virtual reality.

^ab^360° MTG: 360° mirror therapy group.

^ac^TMTG: traditional mirror therapy group.

^ad^MFT: manual function test.

^ae^BBT: box and block test.

^af^ICU: intensive care unit.

^ag^NRS: numeric rating scale.

^ah^PSQI: Pittsburg Sleep Quality Index.

^ai^OT: occupational therapy.

^aj^ARAT: action research arm test.

^ak^FIM: functional independence measure.

^al^PASS-IADL: performance assessment of self-care skills—instrumental activities of daily living.

^am^PASS-BADL: performance assessment of self-care skills—basic activities of daily living.

^an^COPD: chronic obstructive pulmonary disease.

^ao^PSQ: Perception of Stress Questionnaire.

^ap^6MWT: 6-minute walk test.

^aq^FEV1: forced expiratory volume for 1 second.

^ar^HADS-A: Hospital Anxiety and Depression Scale-Anxiety.

^as^HADS-D: Hospital Anxiety and Depression Scale-Depression.

^at^APAIS: Amsterdam Preoperative Anxiety and Information Scale.

^au^LASA: Linear Analog Scale Assessment.

^av^OCCU: Oncology Counseling and Care unit.

^aw^CIMA: Chartered Institute of Management Accountants.

^ax^DDE: emotional discomfort detection.

^ay^Mini-MAC: mini-mental adjustment to cancer.

^az^LAR: low anterior resection.

^ba^TaTME: transanal total mesorectal resection.

^bb^CFS: Cancer Fatigue Scale.

^bc^CES-D: Center for Epidemiological Studies-Depression Scale.

^bd^FACT-leu: functional assessment of cancer therapy-leukemia.

## Discussion

### Principal Findings

We found 24 RCTs of VR for older adults with serious illness that encompassed diverse conditions, such as stroke, cancer, cardiovascular disease, and COPD. Among these studies, VR has been applied to improve anxiety and pain, as measured by the HADS, State Anxiety Inventory, or NPS, or mobility and strength, as measured by the Fugl-Meyer upper extremity assessment, proprioception, or orientation metrics. In this review, short VR experiences were typically used to improve outcomes related to anxiety and pain, while longer ones were used to improve mobility and strength after stroke. Training, supervision, and assistance provided were described in 5 studies [38,40,43,46,54]. Of the included RCTs, all 24 studies addressed older adults; however, only a few focused entirely on the older adult population: 3 studies (12%) included adults with an average age of 66 to 70 years [44,50,56], and only 1 study (4%) included older adults with an average age of >75 years [37]. From the 24 RCTs included in this systematic review, 7 (29%) studies adhered to all 8 gold standard VR consensus standards criteria, and all studies (100%) adhered to at least 5 criteria.

Although there are emerging bodies of evidence on VR use for acute pain, physical therapy, and mental health, we found relatively few studies that met our criteria, focused on symptomatic and functional aspects of serious illness or their use for older adults [59-61]. Acute pain and pain associated with serious illness are distinct, as suggested by the total pain model for serious illness by Saunders; are multifactorial; and explicitly consider physical, emotional, social, and existential factors in worsening symptoms [62,63]. Function is also impaired to a greater degree in older adults, and the average American lives with 1 to 2 ADL disabilities during the last 2 years of life [64]. Comorbidity is common among older adults, and underlying conditions and treatments can interact to exacerbate symptoms [65]. Owing to this, the same interventions used to manage acute pain may not be efficacious for managing pain associated with serious illness, especially among older adults with serious illnesses.

The practicality and efficacy of VR for older patients may be limited by difficulty using the technology, cost, and limited accessibility in medical settings [66]. For example, an approximately 1- to 2-pound VR headset may be relatively heavy, which is challenging for patients with frailty, as well as those with a serious illness [67]. In many cases, older patients may tend to be unfamiliar with new technologies and, therefore, may require supervision and assistance. This role can be fulfilled with caregivers, friends, or clinicians. In addition, immersive VR can cause motion sickness, which is a concern for older adults with serious illnesses, and are costlier than nonimmersive therapies [68]. Unfortunately, 5 (21%) of the 24 assessed studies lacked descriptions of supervision, training, or assistance provided, which may limit the ability of other researchers or providers to translate the results to their settings [45,48,50,51,56]. Therefore, VR assessments in older adults should include whether and how users were trained and supported, how their clinical condition impacted their ability to use the system, and how their ability to independently use VR was verified.

The extent to which VR treatment efficacy is reliant on media content remains unclear. This is particularly important because VR audio and visual media content varies generally within and between different VR systems. Furthermore, different clinical conditions and intervention goals may lead to the use of specific VR resources, interactive functionality, interactivity, and content. As a result, there are fundamental limitations for researchers and clinicians in understanding the mechanisms and efficacy of VR due to the opaqueness or lack of content descriptions in many publications [69]. In addition, subtleties in achieving high presence or in how perceived reality is portrayed may significantly impact the clinical impact and patient satisfaction. Therefore, better-published specifications of VR media, such as soundscapes, public accessibility, and other features, are required to better evaluate and clinically apply VR to patients [11].

This evaluation is limited by the sparse availability of specific VR content that was used in the previous studies. In addition, the outcomes related to patients’ quality of life in the different studies were heterogeneous, and therefore, a quantitative synthesis was not possible. These factors, combined, limited the ability to characterize the effects of specific VR features on patient outcomes. However, this may be a representation of an evolving field with limited reporting guidelines. In addition, this study focused specifically on immersive VR, and future research can further examine the effectiveness of different types of VR. One of the original aims of this study was to compare individual differences in VR effectiveness (eg, by gender, race, and ethnicity); however, this was not possible due to a lack of sufficient detail in included studies. Nonetheless, this systematic review provides researchers and clinicians with a detailed overview of the current state of the literature for older adults with serious illness, as well as an awareness and emphasis on the need to follow standard best practices to enhance VR research rigor. Future directions to build upon this systematic review include evaluating different types of VR media and frequency and duration to target improvements in specific outcomes in patients with serious illness. Future studies may also consider more structured supervision and training for VR use for older adults and determine whether this leads to increased compliance and efficacy of VR to improve outcomes in serious illness. Finally, future research can focus on making VR more accessible to everyone, including older adults [70].

### Conclusions

Our systematic review identified a growing body of research evaluating VR apps for older adults with serious illness through RCTs. The literature review illustrates the potential of VR in this population and finds promising but limited extant evidence. These limitations also highlight an important gap in the development and understanding of using VR to improve health and wellness, which are crucial to address for rapidly aging populations.

## Data Availability

The datasets analyzed during this study are publicly available, as detailed in the Methods section.

## Appendix

Multimedia Appendix 1 Search strategy.

Multimedia Appendix 2 PRISMA (Preferred Reporting Items for Systematic Reviews and Meta-Analyses) checklist.

Multimedia Appendix 3 Abstraction form.

Multimedia Appendix 4 Risk of bias of included studies.

## Abbreviations

ADL: activity of daily living
COPD: chronic obstructive pulmonary disease
HADS: Hospital Anxiety and Depression Scale
PRISMA: Preferred Reporting Items for Systematic Reviews and Meta-Analyses
RCT: randomized controlled trial
VR: virtual reality
