# Supplementary material for: Using Virtual Reality to Improve Outcomes Related to Quality of Life Among Older Adults With Serious Illnesses: Systematic Review of Randomized Controlled Trials
Source: J Med Internet Res. 2025 Feb 26;27:e54452. doi: 10.2196/54452 (PMC11904368; doi:10.2196/54452)
Supplement: Multimedia Appendix 1 [file jmir_v27i1e54452_app1.docx]

**Appendix S1:** Search Strategy

**PubMed (8,766 results) - 05/15/2024**

("virtual reality"[Mesh] OR ((virtual*[tiab] OR simulat*[tiab]) AND (realit*[tiab] OR patient*[tw] OR therapy[sh] OR therap*[tiab]) ) OR haptic[tw] OR Immersive Virtual Environments[tw] OR Collaborative virtual environments[tw] OR Shared virtual environments[tw] OR Immersive learning environments[tw] OR Head-mounted display[tw] OR HMD[tw] OR headset[tw] OR CAVE[tw] OR ("computer simulation"[Majr] NOT ("augmented reality"[Mesh] NOT "virtual reality"[Mesh])) NOT education[Majr])

AND

("randomized controlled trial"[PT] OR "controlled clinical trial"[PT] OR "randomiz*"[tiab] OR "placebo"[tiab] OR "clinical trials as topic"[MH] OR "randomly"[tiab] OR "trial"[ti] OR "clinical trial, phase III"[pt] OR "phase 3"[tw] OR "phase3"[tw] OR "phase III"[TW] OR "P3"[TW] OR "PIII"[TW] OR "random allocation"[MH] OR "double-blind method"[MH] OR "single-blind method"[MH] OR "clinical trial"[pt] OR "clinical trial, phase I"[pt] OR "clinical trial, phase II"[pt] OR "clinical trial, phase IV"[pt] OR "multicenter study"[PT] OR "clinical trial*"[tw] OR placebo*[tw] OR sham[tiab] OR "pilot"[tiab] OR "pilots"[tiab] OR "pilot studies"[tiab] OR "pilot study"[tiab] OR "pilot project"[tiab] OR "pilot projects"[tiab] OR "pilot projects"[MH] )

AND

("critical illness"[Mesh] OR "chronic disease" [Mesh] OR "terminal care"[Mesh] OR "critical care"[Mesh] OR "serious illness"[tw] OR terminal[tw] OR chronic[tw] OR advanced[tw] OR severity[tw] OR severe[tw] OR failure*[tw] OR end stage[tw] OR endstage[tw] OR dying[tw] OR “chronic pain”[mesh] OR Intensive Care Units[MeSH] OR intensive care[tw] OR ICU[tw] OR hospice*[tw] OR terminally ill*[tw] OR cancer*[tw] OR neoplas*[tw] OR carcinoma*[tw] OR malignan*[tw] OR tumor*[tw] OR oncolog*[tw] OR sarcoma*[tw] OR adenocarcinoma[tw] OR metasta*[tw] OR neoplasms[Mesh] OR tumour[tw] OR tumours[tw] OR leukemia*[tw] OR lymphoma[tw] OR melanoma[tw] OR blastoma[tw])

AND "English"[la] AND 2009:2024[dp]

NOT ("animals"[MH] NOT "humans"[MH]) NOT (("infant"[mesh] OR "child"[mesh] OR "adolescent"[mesh] OR pediatrics[Mesh] OR child*[tiab] OR teen*[tiab] OR adolescen*[tiab]) NOT ("adult"[mesh] OR adult*[tw])) NOT (pediatric [tiab] OR pediatric*[ta] OR "case report*"[tw] OR "letter"[PT] OR "case reports"[pt] OR "editorial"[pt] OR "comment"[PT] OR "protocol"[TI] OR "technical note"[TI] OR "technical notes"[TI] OR "technical report"[TI] OR "technical review"[TI] OR "technical solution"[TI] OR quasi[tiab])

**Embase (5200 results) - 05/15/2024**

#12 5,200

#10 AND #11

#11 22,586,250

'english':la AND [2009-2024]/py

#10 6,350

#8 NOT #9

#9 9,140,688

pediatric:ti,ab OR pediatric*/jt OR 'case report*':ti,ab OR 'letter'/de OR 'case report'/de OR 'editorial'/de OR 'note'/de OR 'conference paper'/exp OR 'protocol':ti OR 'technical note':ti OR 'technical notes':ti OR 'technical report':ti OR 'technical review':ti OR 'technical solution':ti OR quasi:ti,ab

#8 9,342

#6 NOT #7

#7 3,257,312

('juvenile'/exp OR 'infant'/exp OR 'child'/exp OR 'pediatrics'/exp OR child*:ti,ab OR teen*:ti,ab OR adolescen*:ti,ab) NOT ('adult'/exp OR adult*:ti,ab,kw)

#6 9,953

#4 NOT #5

#5 6,365,057

'animal'/exp NOT 'human'/exp

#4 10,056

#1 AND #2 AND #3

#3 115,283

('virtual reality'/exp OR 'virtual reality' OR 'virtual reality system'/exp OR 'virtual reality exposure therapy'/exp OR 'virtual reality simulator'/exp OR (((virtual* OR simulat*) NEAR/4 (realit* OR patient* OR therap* OR environment*)):ti,ab) OR haptic:ti,ab,kw OR 'immersive virtual environments':ti,ab,kw OR 'collaborative virtual environments':ti,ab,kw OR 'shared virtual environments':ti,ab,kw OR 'immersive learning environments':ti,ab,kw OR 'head-mounted display*':ti,ab,kw OR hmd:ti,ab,kw OR headset*:ti,ab,kw OR 'cave':ti,ab,kw OR ('computer simulation'/exp/mj NOT ('augmented reality'/exp NOT 'virtual reality'/exp))) NOT 'education'/exp/mj

#2 8,231,137

'randomized controlled trial'/exp OR 'clinical trial (topic)'/exp OR 'clinical trial'/exp OR 'randomiz*':ti,ab OR 'placebo':ti,ab OR 'randomly':ti,ab OR 'trial':ti OR 'phase 1 clinical trial'/exp OR 'phase 3':ti,ab,kw OR 'phase3':ti,ab,kw OR 'phase iii':ti,ab,kw OR 'p3':ti,ab,kw OR 'piii':ti,ab,kw OR 'randomization'/exp OR 'double blind procedure'/exp OR 'single blind procedure'/exp OR 'triple blind procedure'/exp OR 'phase 2 clinical trial'/exp OR 'phase 3 clinical trial'/exp OR 'phase 4 clinical trial'/exp OR 'major clinical study'/exp OR 'multicenter study'/exp OR 'clinical trial*':ti,ab,kw OR placebo*:ti,ab,kw OR sham:ti,ab OR 'pilot':ti,ab OR 'pilots':ti,ab OR 'pilot studies':ti,ab OR 'pilot study':ti,ab OR 'pilot project':ti,ab OR 'pilot projects':ti,ab OR 'pilot study'/de

#1 15,020,647

'critical illness'/exp OR 'chronic disease'/exp OR 'terminal disease'/exp OR 'long term care'/exp OR 'intensive care'/exp OR 'serious illness':ti,ab,kw OR terminal:ti,ab,kw OR chronic:ti,ab,kw OR advanced:ti,ab,kw OR severity:ti,ab,kw OR severe:ti,ab,kw OR failure*:ti,ab,kw OR 'end stage':ti,ab,kw OR endstage:ti,ab,kw OR dying:ti,ab,kw OR 'chronic care':ti,ab,kw OR 'chronic pain'/exp OR 'intensive care unit'/exp OR 'intensive care':ti,ab,kw OR icu:ti,ab,kw OR hospice*:ti,ab,kw OR 'terminally ill*':ti,ab,kw OR cancer*:ti,ab,kw OR neoplas*:ti,ab,kw OR carcinoma*:ti,ab,kw OR malignan*:ti,ab,kw OR tumor*:ti,ab,kw OR oncolog*:ti,ab,kw OR sarcoma*:ti,ab,kw OR adenocarcinoma:ti,ab,kw OR metasta*:ti,ab,kw OR 'neoplasm'/exp OR tumour:ti,ab,kw OR tumours:ti,ab,kw OR leukemia*:ti,ab,kw OR lymphoma:ti,ab,kw OR melanoma:ti,ab,kw OR blastoma:ti,ab,kw

**CINAHL (788 results) - 05/15/2024**

S12 788

S10 AND S11

S11 6,160,467

LA"english" AND PY"2009-2024"

S10 914

S8 NOT S9

S9 504,592

( TI”pediatric*” OR AB”pediatric*” OR SO”pediatric*” OR MH"case studies" OR TI”case report*” OR TI”case stud*” OR AB”case report*” OR AB”case stud*” OR TI"protocol" OR TI"technical note" OR TI"technical notes" OR TI"technical report" OR TI"technical review" OR TI"technical solution" OR TI”quasi” OR AB”quasi” )

S8 1,020

S6 NOT S7

S7 825,542

(MH“child+” OR MH”minors” OR MH”adolescence+” OR MM”pediatrics” OR TI(child* OR teen* OR adolescen*) OR AB(child* OR teen* OR adolescen*) ) NOT (MH“adult+” OR adult*)

S6 1,091

S4 NOT S5

S5 87,217

(MH“animals” NOT MH”human”)

S4 1,092

S1 AND S2 AND S3

S3 32,452

(((virtual* OR simulat*) N4 (realit* OR patient* OR therap* OR environment*)) OR MH“virtual reality+” OR ”virtual reality” OR MH”virtual reality exposure therapy” OR haptic OR ”immersive virtual environment*” OR ”collaborative virtual environment*” OR ”shared virtual environment*” OR ”immersive learning environment*” OR ”head-mount*” OR hmd OR "headset*” OR ”cave” OR (MM“computer simulation+” NOT (MH“augmented reality” NOT MH”virtual reality”))) NOT MM”education”

S2 634,287

MH“randomized controlled trials+” OR MH”clinical trials+” OR TI(”randomiz*” OR ”placebo” OR ”randomly”) OR AB(”randomiz*” OR ”placebo” OR ”randomly”) OR TI”trial” OR ”phase 3” OR ”phase3” OR ”phase iii” OR ”p3” OR ”piii” OR MH”random sample+” OR MH”double-blind studies” OR ”MH”single-blind studies” OR MH”triple-blind studies” OR ”major clinical stud” OR ”multicenter stud*” OR ”clinical trial*” OR placebo* OR sham OR TI(”pilot” OR ”pilots”) OR AB(”pilot” OR ”pilots”) OR MH”pilot studies” OR ”pilot stud*” OR ”pilot project” OR ”pilot projects”

S1 2,051,425

MH“critical illness” OR MH”chronic disease” OR MH”terminal ill patients” OR MH”critical care” OR MH”long term care” OR MH”terminal care” OR “serious illness” OR terminal OR chronic OR advanced OR severity OR severe OR failure* OR “end stage” OR endstage OR dying OR “chronic care” OR MH”chronic pain” OR MH“intensive care units” OR “intensive care” OR icu OR hospice* OR “terminally ill*” OR cancer* OR neoplas* OR carcinoma* OR malignan* OR tumor* OR oncolog* OR sarcoma* OR adenocarcinoma OR metasta* OR MH”neoplasms” OR tumour OR tumours OR leukemia* OR lymphoma OR melanoma OR blastoma
