# Supplementary material for: Using Virtual Reality to Improve Outcomes Related to Quality of Life Among Older Adults With Serious Illnesses: Systematic Review of Randomized Controlled Trials
Source: J Med Internet Res. 2025 Feb 26;27:e54452. doi: 10.2196/54452 (PMC11904368; doi:10.2196/54452)
Supplement: Multimedia Appendix 3 [file jmir_v27i1e54452_app3.docx]

**Appendix S2:** Abstraction Form

**Identification**

**Study Details**

Sponsorship source: *The source of funding for this project.*

Country:

Setting:

Publication date:

**Author’s contact details**

Author’s name:

Institution:

Email:

Address:

**Methods**

Design:

**Population**

Inclusion Criteria: *Write out the study’s inclusion criteria for participants*

Exclusion Criteria: *Write out the study’s exclusion criteria for participants*

Group Differences: *If no differences, type “N/A” (because the studies should be randomized controlled trials, there likely will be no significant difference within the groups that impact the analysis of the outcomes).*

**Baseline Characteristics**

| **Characteristics** | **Intervention Arm** | **Control** | **Overall** |
| --- | --- | --- | --- |
| **Sample Size** (N) |  |  |  |
| **Gender** | N male (%)  N female (%)  N other (%) | N male (%)  N female (%)  N other (%) | N/A |
| **Age**  Mean (SD); Range |  |  | N/A |
| **Race/Ethnicity** | American Indian/Alaska Native: N (%)    Asian: N (%)    Black or African American:  N (%)    Native Hawaiian/Other Pacific Islander: N (%)    Hispanic or Latino: N (%)    White: N (%) |  | N/A |
| **Type of serious illness(s)** | *What type of serious illness are the participants of the study suffering from (eg. dementia, stroke, cancer, etc…)?* |  |  |

**Interventions**

Overall Directions: *Some boxes will not be applicable to certain studies, and thus can be filled in with “N/A.”*

| **Question** | **Intervention Arm** | **Control** |
| --- | --- | --- |
| **Arm description** | *Copy paste all the details included in the article* | *Copy paste all the details included in the article* |
| **Setting** | *Where is the* ***intervention*** *taking place (eg. in-patient/hospital, clinic based/ambulatory, skilled nursing facilities, home, other)?* |  |
| **Follow up timepoints and duration** | *At what time points were patient reported outcomes collected (eg. baseline, 3 months, 6 months, etc…)?* |  |
| **Was there training in the use of VR?** | *Did a researcher, nurse, expert etc. have to teach the patient to use VR prior to VR intervention?*  *or was there a form of virtual training program? Or not enough information to determine.* |  |
| **Was there supervised use of VR?** | *Supervision definition= watching to make sure harm didn’t happen, make sure they are doing it correctly, troubleshoot with issues that come up.*  *If it was supervised, what did the supervisor do? Or not enough information to determine.* |  |
| **Who assisted patients with using the VR headset?** | *Researchers, hospital staff, family members/ caregivers, etc. Or not enough information to determine.* |  |
| **What was the purpose of the Virtual Reality intervention?** | *Eg. Range of motion improvement? Calming purposes? Cognitive training? Pain therapy? Distraction from pain?* |  |
| **What was the VR headset type?** | *Body sensors, hand-held or gaze controlled, or “not described”*  *Was the VR model specified?* |  |
| **What was the media content used in the study?** | *360 videos? Pre-produced videos? Were games involved?*  *Also include the subject of the content (is it a fighting game, is it a sports game, etc…)* |  |
| **Duration of session, frequency of session, duration of the VR intervention** | *Eg. 2 hours, twice a day for 3 weeks* |  |
| **Was VR used in combination with another treatment?** | *If yes, what treatment?*  *What was the therapeutic context of VR?* |  |
| **Was there a social VR component?** | *Were they interacting with others through VR? Therapeutic group, PTSD?*  *Who was it with?* |  |
| **What were the patient functional outcomes?** | *List all the patient quality outcomes* | *List all the patient quality outcomes* |
| **Did patient functional outcomes improve?** | *If yes, list which outcomes improved and designate if significant improvement.*  *If not, list the major outcomes that were measured and indicate there was no significant improvement.* | *If yes, list which outcomes improved and designate if significant improvement.*  *If not, list the major outcomes that were measured and indicate there was no significant improvement.* |
| **Was the trial reported with the appropriate registries? If so, which one?** | *Write the registry the trial is reported with and write the registration number.* |  |
| **Did the study include VA sites or patients?** |  |  |

**Outcomes**

Overall Directions: Each study will likely have different outcomes and different ways they are reporting the outcomes, thus, individual tables will have to be built for each article (some articles might have multiple usable outcomes and will have multiple tables that we will extract).

- Outcome name: *Include the different types of patient reported outcomes (quality of life, depressive symptoms, anxiety symptoms, etc…).*
- Outcome type: Continuous, Dichotomous, Adverse event
- Reported as: Confidence intervals (mean, CI, N), Standard deviation (mean, SD, N), Standard Error (mean, SE, N)
- Outcome group: *usually not applicable*
- Scale: *enter the name of scale, i.e FACT-G*
- Range: *enter the possible range of results, i.e. 0-10*
- Unit of measurement: *usually not applicable*
- Direction*:* Lower is better or Higher is better
- Data value: Is the change calculated from baseline or endpoint (*usually change is calculated from baseline*)
- Notes: *anything else that helps us better understand the results*
