# Supplementary material for: Using Virtual Reality to Improve Outcomes Related to Quality of Life Among Older Adults With Serious Illnesses: Systematic Review of Randomized Controlled Trials
Source: J Med Internet Res. 2025 Feb 26;27:e54452. doi: 10.2196/54452 (PMC11904368; doi:10.2196/54452)
Supplement: Multimedia Appendix 4 [file jmir_v27i1e54452_app4.docx]

**Appendix S3:** Risk of Bias of Included Studies

| **Study** | **Sequence Generation** | **Allocation Concealment** | **Blinding of Participants and Personnel** | | **Blinding of Outcome Assessors** | **Incomplete Outcome Data** | **Selective Outcome Reporting** | | **Other Source of Bias** | **Overall Risk of Bias** |
| --- | --- | --- | --- | --- | --- | --- | --- | --- | --- | --- |
| Bani Mohammad and Ahmad, 2018 | Low | Low | Low | High | | Low | Low | Low | | High |
| Burrai et. al, 2023 | Low | Low | Low | High | | Low | Low | Low | | High |
| Chatterjee et al., 2022 | Low | Low | High | Low | | Low | Low | Low | | High |
| Chirico et al., 2020 | Low | High | High | Unclear | | Low | Low | Low | | High |
| Groninger et al., 2021 | Low | Low | High | Low | | Low | Low | Low | | High |
| Hsu et al., 2022 | Low | Low | Low | Low | | Low | Low | Low | | Low |
| Huang et al., 2022 | Low | Low | Low | Low | | Low | Low | Low | | Low |
| Huang et al, 2024 | Low | Low | Low | High | | Low | Low | Low | | High |
| Jo et al., 2024 | Low | Low | Low | Low | | Low | Low | Low | | Low |
| Laghlam et al., 2021 | Low | Low | High | Unclear | | Low | Low | Low | | High |
| Lee and Kang, 2020 | Low | Low | High | Unclear | | Low | Low | Low | | High |
| Mekbib et al., 2021 | Low | Low | High | Low | | Low | Low | Low | | High |
| Menekli et al., 2022 | Low | Low | High | High | | Low | Low | Low | | High |
| Ögün et al., 2019 | Low | Low | Low | Unclear | | Low | Low | Unclear | | Low |
| Park et al., 2013 | Low | Low | High | Unclear | | Low | Low | Low | | High |
| Shin et al., 2023 | Low | Low | Low | Low | | Low | Low | Low | | Low |
| Spiegel et al., 2019 | Low | Low | Low | Low | | Low | Low | Low | | Low |
| Song et al., 2021 | Low | Low | Unclear | Unclear | | Low | Low | Low | | Low |
| Torres García et al., 2023 | Low | Low | Low | High | | Low | Low | Low | | High |
| Turrado et al., 2021 | Low | High | High | Unclear | | Low | Low | Low | | High |
| Rousseaux et al., 2022 | Low | Low | High | Unclear | | Low | Low | Low | | High |
| Rutkowski et al., 2021 | Low | Low | Low | Low | | Low | Low | Low | | Low |
| Uslu et al., 2023 | Low | Low | Low | High | | Low | Low | Low | | High |
| Zhang et al., 2024 | Low | Low | Low | High | | Low | Low | Low | | High |
